# Use of vascularised cartilage as an additional interposition in temporomandibular ankylosis surgery: Rationale, advantages and potential benefits

**DOI:** 10.4103/0970-0358.44708

**Published:** 2008

**Authors:** Mukund Jagannathan, Maksud Devale, Prashantha Kesari, Siddharth Karanth

**Affiliations:** Department of Plastic and Reconstructive Surgery, Lokmanya Tilak Muncipal Medical College and Hospital, Sion, Mumbai-400 022, India

**Keywords:** Temporomandibular joint ankylosis, vascularised cartilage flap

## Abstract

**Context:**

Surgery for the release of temporomandibular joint (TMJ) ankylosis is a commonly performed procedure. Various interposition materials have been tried with varying success rates. However, none of these procedures attempt to recreate the architecture of the joint as the glenoid surface is usually left raw.

**Aims:**

We aimed to use a vascularised cartilage flap and to line the raw surface of the bone to recreate the articular surface of the joint.

**Settings and Design:**

There is a rich blood supply in the region of the helical root, based on branches from the Superficial Temporal Artery (STA), which enables the harvest of vascularised cartilage from the helical root for use in the temporomandibular joint.

**Materials and Methods:**

Two cases, one adult and the other a child, of unilateral ankylosis were operated upon using this additional technique. The adult patient had a bony segment excised along with a vascularised cartilage flap for lining the glenoid. The child was managed with an interposition graft of costochondral cartilage following the release of the ankylosis, in addition to the vascularised cartilage flap for lining the glenoid.

**Results:**

The postoperative mouth opening was good in both the cases with significant reduction in pain. However, the long-term results of this procedure are yet to be ascertained.

**Conclusions:**

The vascularised cartilage flap as an additional interposition material in temporomandibular joint surgery enables early and painless mouth-opening with good short-term results. The potential applicability of this flap in various pathologies of the temporomandibular joint is enormous.

## INTRODUCTION

Temporomandibular joint (TMJ) ankylosis needs aggressive management consisting of several parts:

- Release of the restraining forces
- Management of the defect created
- Postoperative physiotherapy

While several methods of interposition arthroplasty have been described, there are very few attempts to recreate the ‘milieu interior’ of the joint. We have attempted to address this issue by means of a pedicled, vascularised cartilage flap. The anatomy of the superficial temporal artery (STA) and its supply to the helical root has been well described by Park *et al.*[1] Based on this blood supply through the STA, several authors have described reverse-flow, composite chondrocutaneous flaps for the reconstruction of the eyelid and the nose.[2–5] These clearly indicate the rich blood supply of the region of the helical root based on branches from the STA, which enables the harvest of vascularised cartilage from the helical root for use in the TMJ. If necessary, the temporoparietal fascia (TPF) can be harvested as an extension based on the STA for additional use. This flap is used to line the raw surface of the glenoid fossa in an attempt to provide a cartilaginous surface, against which the condyle can move. By this manoeuvre, we hope that a more physiological joint can be recreated.

## MATERIALS AND METHODS

In this preliminary study, two cases of unilateral ankylosis, one an adult and the other a child, were operated on using this additional technique. The adult patient had a bony segment excised along with a vascularized cartilage flap for lining the glenoid. The child was managed with an interposition graft of costochondral cartilage following the release of ankylosis, in addition to the vascularized cartilage flap for lining the glenoid.

## TECHNIQUE

**Figure 1:**
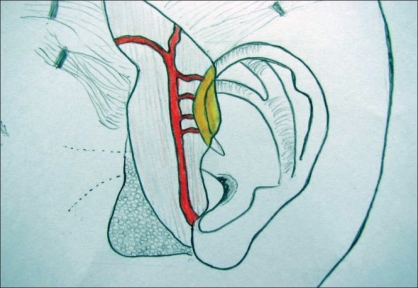
Diagram showing branches of STA to helical root

**Figure 2:**
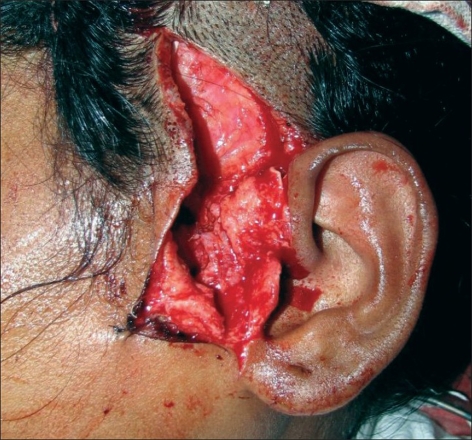
Initial dissection to harvest cartilage preserving branches to helical root

**Figure 3:**
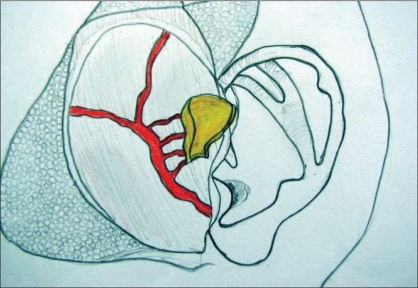
Diagram of harvested cartilage with cuff of TPF prior to harvesting

**Figure 4:**
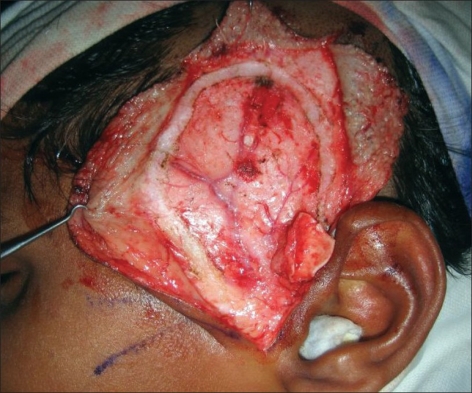
Photo showing harvested cartilage with cuff of TPF

**Figure 5:**
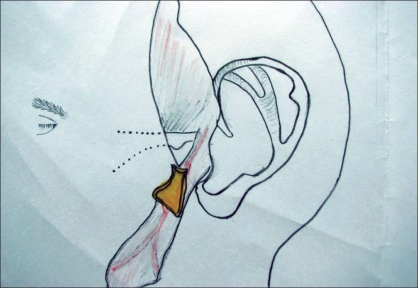
Diagram of composite flap showing vascularised cartilage and TPF, based on the STA

**Figure 6:**
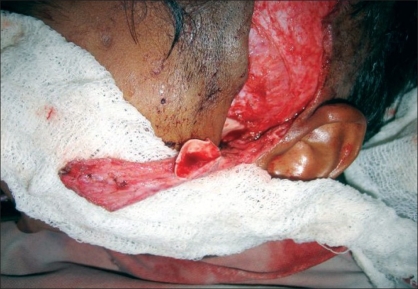
Photo showing composite flap consisting of harvested cartilage and TPF

The incision for exposure of the TMJ is taken just anterior to the ear, but not deepened. Subdermal dissection is performed posteriorly in the region of the root of the helix, elevating the auricular skin off the cartilage in an extraperichondrial fashion [Figures 1 and 2]. The dissection is continued around the free border of the helical rim and into the cymba conchae. The vertical dimension of the harvested cartilage is around 1.5 cm. An adequate width of cartilage is taken and the flap is elevated along with a generous cuff of soft tissue, presumably containing the branches from the STA to the helix [Figures 3 and 4]. The skin incision is extended superiorly and anteriorly, and dissection is again performed in the subfollicular plane to expose and harvest a portion of the TPF. The STA is ligated at the upper end and the flap is dissected down till the level of the tragus [Figures 5 and 6]. Protecting the pedicle, the incision is deepened through the deep temporal and parotido- masseteric fascia, which are reflected anteriorly (the technique of subfascial exposure of the TMJ) till the abnormal pathology is delineated. The ankylosis is dealt with in a standard fashion, creating a gap followed by a coronoidectomy. Masseter release and soft tissue release complete the initial procedure.

**Figure 7:**
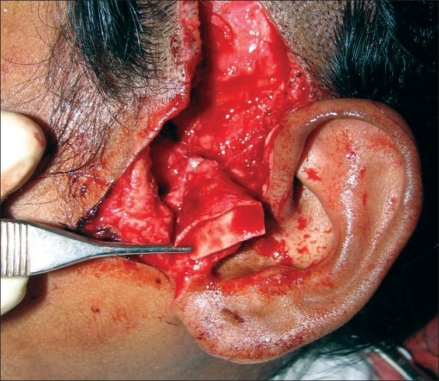
Excellent bleeding from the cartilage

**Figure 8:**
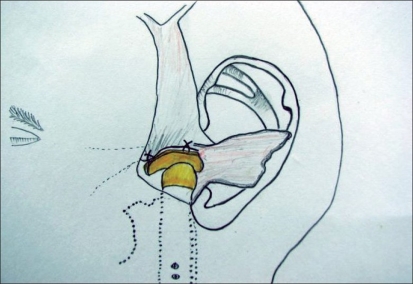
Diagram of vascularised cartilage flap lining the glenoid cavity, with the costochondral graft in situ

**Figure 9:**
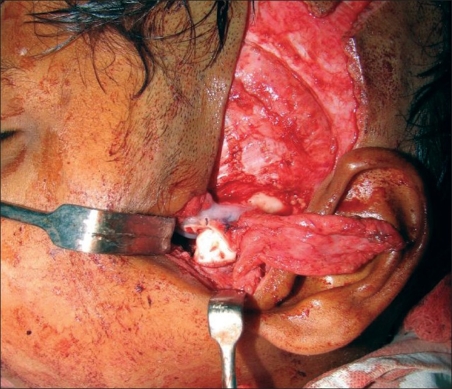
Photo showing the cartilage lining the glenoid, retained with fibrin glue and sutures, with the costochondral graft in place, showing the formation of the joint

The vascularized cartilage flap showing good bleeding from the surface [Figure 7] is then placed in the glenoid with the cartilage cup facing downwards. Fibrin glue is used to retain the flap in position, which is further reinforced with a couple of stitches near the root of the zygoma. The costochondral graft (if indicated) is fixed to the ramus. The condylar end should abut comfortably against the cartilage cup [Figures 8 and 9] and the mouth should be repeatedly opened and closed to ensure that the interposed cartilage is stable even during movement of the condyle. (**Video clip can be seen on www.ijps.org**) The TPF can be used to wrap around the upper end of the graft as an additional vascular bolster for the cartilage end of the graft; closure is routine.

Postoperative jaw opening exercises were instituted after 48 hours. The adult patient has been on regular follow-up for six months and the child for a month as this article is being drafted.

## RESULTS

**Figure 10:**
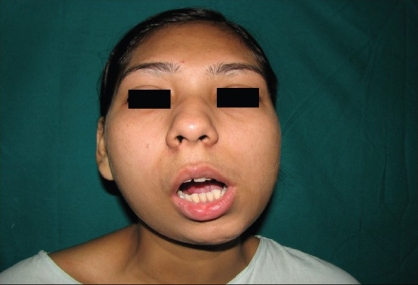
The preoperative picture of the adult patient

**Figure 11:**
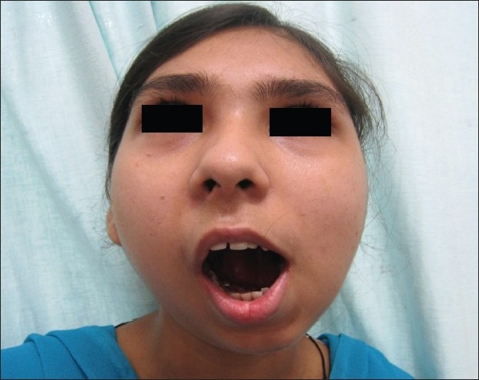
The postoperative picture of the adult patient at six months follow-up

The postoperative recovery has been uneventful in both the cases. A significant finding was a reduction in pain on opening of the mouth in the early postoperative period. Both the patients have adequate and stable mouth opening [Figures 10 and 11]. There is no significant deformity of the ears; the long-term results are to be ascertained.

## DISCUSSION

Management of the defect following resection of the ankylosed joint is the central feature of the surgical management of TMJ ankylosis. Gap arthroplasty, a common method of treatment, has many disadvantages. In a controlled study in sheep, Matsuura *et al.*[6] showed that even after three months, the residual fibrous tissue in the gap is poorly organised and has deposition of bone within it. They concluded that the picture simulates a fibrous reankylosis, and that the mouth opening gradually reduces over a period of time. Cheung *et al.*[7] performed gap arthroplasty along with distraction to simultaneously achieve lengthening of the ramus, along with transport of the pseudocondyle into the glenoid fossa. In their series of five cases, they showed adequate mouth opening after one year, and postulated that the disorganized fibrous tissue in the gap was compressed into an organized layer, simulating the disc of the joint.

Roychowdhury *et al.*[8] have shown a series of 50 cases treated by gap arthroplasty and intensive postoperative jaw exercises to have a recurrence of ankylosis in only two cases.

However, interposition arthroplasty appears to be the mainstay of treatment and various materials have been used. Acrylic,[9,10] dermis fat graft,[11] muscle or myofascial flaps,[12] auricular cartilage (nonvascularized),[13] resected bony material,[14,15] and costochondral grafts in different forms[16–18] have all been used with varying degrees of success. Costochondral grafts have been shown to adaptively grow with downward growth of the maxilla and mandible, thus maintaining vertical height. Even the buccal pad of fat has been used as an interposition.[19]

However, in all these techniques, no attention is paid to the true lining of the glenoid cavity with a suitable material. The role of the articular disc was stressed and several authors have repositioned the disc to act as a cushion for the condyle.[20–22] They have stated that this manoeuvre goes a long way in preventing reankylosis and have demonstrated consistent long-term results. In fact, Takaishi *et al.*[23] have conducted a study using nonvascularised auricular cartilage in sheep. Histological examination of the cartilage after three months revealed a viable cartilage with adequate joint space between the cartilage and the glenoid.

We believe that one of the inadequately addressed issues in TMJ ankylosis management is the providing of a vascular ‘cushion’ to line the raw surface of the glenoid following resection of the ankylosed mass. This has the following advantages:

- Provides a cartilage lining for the condylar remnant or the graft to move against.
- Augments the vertical height of the ramus.
- Reduces the pain of movement in the immediate postoperative period.

**In addition, there may be some potential benefits:**

- Additional cartilage deposition in response to stress as the cartilage is covered with perichondrium on both sides and retains its vascularity.
- Additional vascularity provided to the costochondral graft by the TPF wrapped around it.

However, this will require a long-term study regarding the fate of such vascularised cartilage flaps.

**Figure 12:**
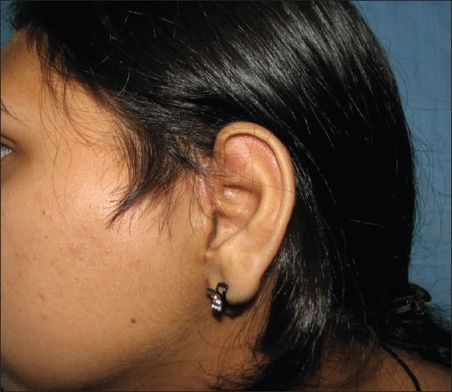
Minimal donor morbidity at the root of the helix

Although the donor site morbidity is minimal, [Figure 12] there is an increase in operative time of around 45 minutes, and it cannot be used in recurrent cases where the delicate vasculature supplying the root of the helix may have been damaged.

## CONCLUSIONS

The vascularised cartilage flap as an additional interposition material in TMJ surgery is a useful addition. It has some tangible benefits and has enormous potential. The use of this technique can be extended to all cases of TMJ reconstruction following trauma, ablative surgery, or craniofacial microsomia. A long-term evaluation of these patients is needed to ascertain whether these potential advantages are realised.
